# Complete genome sequence of a circulating foot-and-mouth disease virus serotype A isolated from recent outbreaks in Bangladesh

**DOI:** 10.1128/mra.01143-25

**Published:** 2026-02-27

**Authors:** Sajedul Hayat, Mohammad Sadekuzzaman, Safeth Arju, Mahbubul Pratik Siddique, Md. Ariful Islam, Md. Golam Azam Chowdhury, Muhammad Tofazzal Hossain, Md. Alimul Islam

**Affiliations:** 1 Department of Microbiology and Hygiene, Bangladesh Agricultural University 54492, Mymensingh, Bangladesh; 2 Central Disease Investigation Laboratory, Department of Livestock Services, Dhaka, Bangladesh; Queens College Department of Biology, Queens, New York, USA

**Keywords:** genome, FMD virus, serotype A, BDSH_01, Bangladesh

## Abstract

We present the complete genome sequence of foot-and-mouth disease virus serotype A isolate BDSH_01 from Bangladesh. The 8,140-nucleotide genome encodes a 2,333-aa polyprotein with conserved motifs. Phylogenetically, within lineage A/ASIA/G-VII, clustering with recent regional strains indicates transboundary transmission. Sequence divergence from vaccine strains highlights its epidemiological and immunological significance.

## ANNOUNCEMENT

Foot-and-mouth disease (FMD) affects even-toed ungulates and significantly impacts the agricultural sector, causing substantial economic losses worldwide (1). The causal agent of this devastating transboundary disease is a non-enveloped, single-stranded, positive-sense RNA virus of the genus *Aphthovirus* within the family *Picornaviridae* (2). In Bangladesh, FMD remains endemic, with serotype O predominating, followed by A and Asia-1 (3). Molecular characterization of circulating isolates is crucial for understanding viral evolution, tracking transboundary spread, and optimizing vaccine selection.

Here, we describe the complete genome of an FMDV serotype A isolate, BDSH_01, collected from an infected bull in July 2024 at Noakhali (22.867000°N, 91.296743°E) in Bangladesh. Viral RNA was extracted from tongue epithelial tissue using a KingFisher mL Purification System with the MagMAX Viral RNA Isolation Kit, according to the manufacturer’s guidelines. Verso cDNA Synthesis Kit (Thermo Scientific) was used for cDNA synthesis. After quality check of the cDNA, the library was prepared using the Rapid Plus DNA Lib Prep Kit for Illumina, including end repair, A-tailing, adapter addition, PCR amplification, fragment screening, and purification. Paired-end sequencing was performed on the Illumina NovoSeq 6000 using the PE150 platform by Novogene (Beijing, China), yielding 15,845,096 high-quality paired-end reads. Genome assembly and annotation were performed on the Galaxy Europe platform (4), achieving 193× coverage. The raw fastq reads were processed through a workflow that included quality trimming with Fastp v1.0.1 (5), reference-guided mapping with Bowtie2 v2.5.4 (6), *de novo* assembly with SPAdes v4.1.0 (7), assembly patching with RagTag v2.1.0 (8), and final annotation with Prokka v1.14.6 (9). Default parameters were used for all software unless otherwise specified. Phylogenetic analysis and amino acid comparison of the VP1 peptide were performed using MEGA11 (10).

The complete genome was 8,140 nucleotides long with a GC content of 54.1%. The genome features a 5′ untranslated region (UTR) of 1,016 nt including a 17-nt poly(C) tract, a single open reading frame encoding a 2,333-amino acid polyprotein, and a 3′ UTR of 114 nt followed by a poly(A) tail of ≥32 nt. The key motifs, including 2A NPGP, the integrin-binding RGD in VP1, were identified. Structural proteins (VP1–VP4) and non-structural proteins (Lpro, 2A–2C, 3A–3D) were annotated using Geneious Prime (v2025.1.3) based on reference sequences. Phylogenetic analysis of the VP1 peptide placed BDSH_01 within the A/ASIA/G-VII lineage, clustering closely with recent isolates from neighboring countries (3, 11), suggesting ongoing cross-border transmission rather than circulation of older local strains.

**Fig 1:**
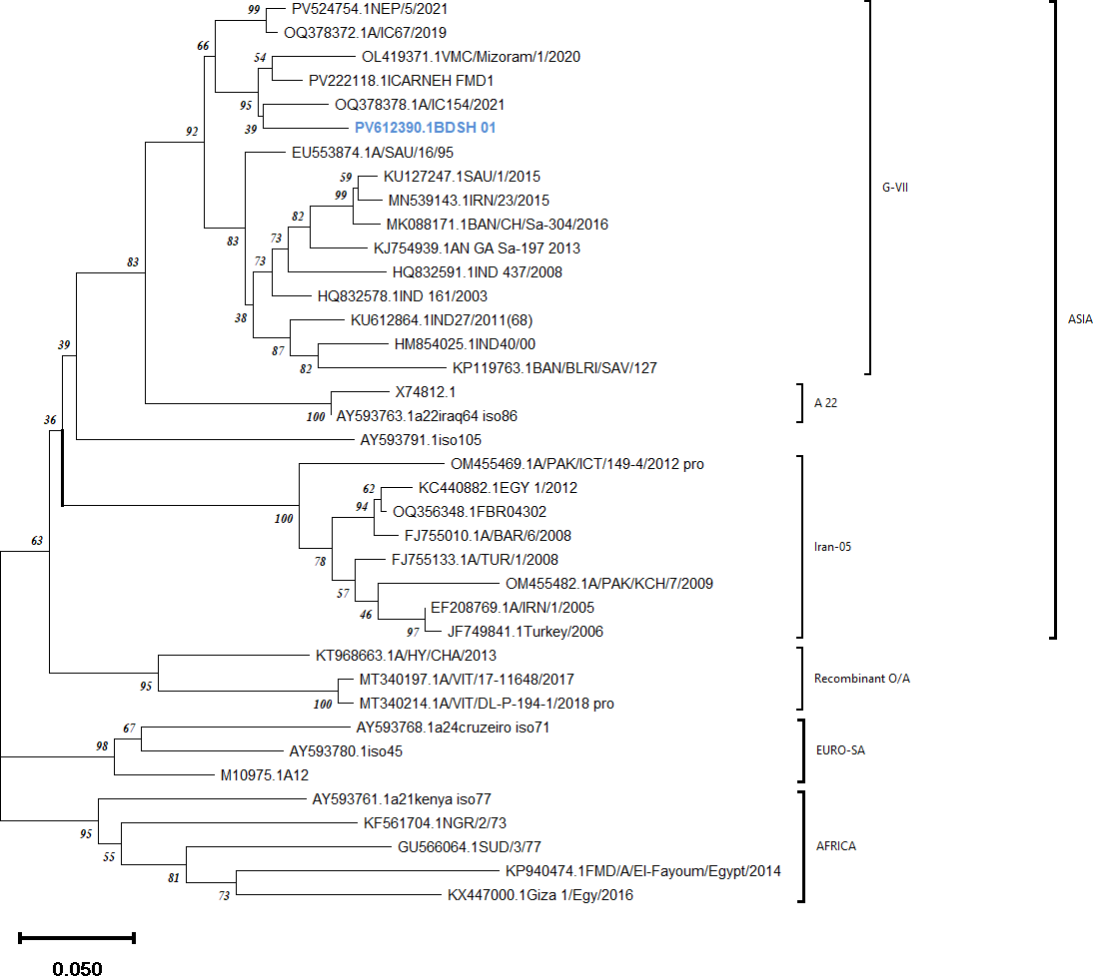
A bootstrap consensus phylogenetic tree, inferred from 1,000 replicates using the neighbor-joining method in MEGA11, represents the evolutionary history of the analyzed taxa.

Crucially, the isolate had significant genetic divergence from existing vaccine strains. BLAST analysis (12) revealed a maximum nucleotide identity of 92.70% with a global isolate (PV524754). Compared to an Indian (HM854025.1) and proposed Bangladeshi (MK088171.1) vaccine strains (13), the identity was 91.18% and 91.06% (Fig. 1). Furthermore, the VP1 region contained four amino acid substitutions (N139S, K140Q, S142F, and I159V/A148T) in the antigenically critical GH loop, which may influence vaccine-induced immunity.

We report the complete genome of FMDV serotype A isolate BDSH_01 from Bangladesh. The data highlight genetic divergence from available vaccine strains and emphasize the importance of molecular epidemiology for the FMD control strategy in the region.

## Data Availability

The complete genome sequence of foot-and-mouth disease virus serotype A isolate BDSH_01 has been submitted to the NCBI GenBank database under BioProject number PRJNA1244571, BioSample number SAMN47709322, SRA number SRR32930079, and accession number PV612390.
